# Agonist-specific voltage-dependent gating of lysosomal two-pore Na^+^ channels

**DOI:** 10.7554/eLife.51423

**Published:** 2019-12-11

**Authors:** Xiaoli Zhang, Wei Chen, Ping Li, Raul Calvo, Noel Southall, Xin Hu, Melanie Bryant-Genevier, Xinghua Feng, Qi Geng, Chenlang Gao, Meimei Yang, Kaiyuan Tang, Marc Ferrer, Juan Jose Marugan, Haoxing Xu

**Affiliations:** 1 Department of Molecular, Cellular, and Developmental BiologyUniversity of MichiganAnn ArborUnited States; 2 Collaborative Innovation Center of Yangtze River Delta Region Green PharmaceuticalsZhejiang University of TechnologyHangzhouChina; 3 National Center for Advancing Translational Sciences (NCATS)Medical Center DriveRockvilleUnited States; 4 Department of NeurologyThe Fourth Hospital of Harbin Medical UniversityHarbinChina; Universidad Nacional Autónoma de MéxicoMexico; The University of Texas at AustinUnited States

**Keywords:** lysosomal two-pore channels, tri-cyclic antidepressants, whole-endolysosome patch clamp, lysosomal Na+ channels, None

## Abstract

Mammalian two-pore-channels (TPC1, 2; *TPCN1, TPCN2*) are ubiquitously- expressed, PI(3,5)P_2_-activated, Na^+^-selective channels in the endosomes and lysosomes that regulate luminal pH homeostasis, membrane trafficking, and *Ebola* viral infection. Whereas the channel activity of TPC1 is strongly dependent on membrane voltage, TPC2 lacks such voltage dependence despite the presence of the presumed ‘S4 voltage-sensing’ domains. By performing high-throughput screening followed by lysosomal electrophysiology, here we identified a class of tricyclic anti-depressants (TCAs) as small-molecule agonists of TPC channels. TCAs activate both TPC1 and TPC2 in a voltage-dependent manner, referred to as Lysosomal Na^+^ channel Voltage-dependent Activators (LyNa-VAs). We also identified another compound which, like PI(3,5)P_2_, activates TPC2 independent of voltage, suggesting the existence of agonist-specific gating mechanisms. Our identification of small-molecule TPC agonists should facilitate the studies of the cell biological roles of TPCs and can also readily explain the reported effects of TCAs in the modulation of autophagy and lysosomal functions.

## Introduction

Lysosomes are the cell’s recycling center equipped with the most important nutrient-sensing machinery in the cell (Lawrence and Zoncu, 2019; Li et al., 2019). Ion channels in the lysosome play essential roles in the regulation of various lysosomal functions, including cargo import, lysosomal degradation, and catabolite export (Li et al., 2019; Xiong and Zhu, 2016). Patch-clamp studies of isolated lysosomal membranes have recently discovered multiple lysosomal channels that are selective for Na^+^, K^+^, Ca^2+^, and Cl^-^ (Cang et al., 2015; Cang et al., 2013; Cao et al., 2015; Dong et al., 2010; Li et al., 2019; Wang et al., 2012; Xiong and Zhu, 2016). How these lysosomal channels are activated by endogenous nutrient-dependent signals remain largely unknown (Li et al., 2019). On the other hand, membrane-permeable small-molecule modulators, that is synthetic agonists and inhibitors, have proved extremely helpful in probing the cell biological functions of intracellular channels, including lysosomal channels (Cao et al., 2015; Dong et al., 2010; Li et al., 2019; Wang et al., 2012; Xiong and Zhu, 2016). For example, small-molecule synthetic agonists of Mucolipin TRP channels (TRPMLs), that is ML-SAs, have been instrumental in revealing the functions of these Ca^2+^ release channels in lysosomal exocytosis, mobility, and biogenesis (Li et al., 2016; Shen et al., 2012; Zhang et al., 2016). However, such chemical tools are still lacking for most other lysosomal channels.

Two-pore channel proteins (TPC1, 2; *TPCN1, TPCN2*) are ubiquitously expressed, dimeric, two-repeat (2 × 6 TM) cation channels that are localized exclusively in the intracellular endosomes and lysosomes (Calcraft et al., 2009; Grimm et al., 2017; Morgan et al., 2011). At the cellular level, TPCs regulate organellar membrane excitability, membrane trafficking, and pH homeostasis; at the organismal level, TPCs regulate various physiological and pathological processes, including hair pigmentation, *Ebola* viral infection, and cancer growth (Ambrosio et al., 2016; Nguyen et al., 2017; Sakurai et al., 2015). Early works from several laboratories suggested that TPCs play an essential role in mediating Ca^2+^ release from endolysosomes in response to cytosolic increases of nicotinic acid adenine dinucleotide phosphate (NAADP) (Brailoiu et al., 2009; Calcraft et al., 2009; Ruas et al., 2010; Zong et al., 2009). However, it remains controversial whether TPCs are the *bona fide* NAADP receptor (Lin-Moshier et al., 2012; Morgan et al., 2015; Walseth et al., 2012). Indeed, several recent endolysosomal patch-clamp studies have demonstrated that TPCs are Na^+^-selective channels activated by PI(3,5)P_2_ (Bellono et al., 2016; Cang et al., 2014; Cang et al., 2013; Guo et al., 2017; Jha et al., 2014; Kirsch et al., 2018; She et al., 2019; Wang et al., 2012), a late endosome and lysosome-specific phosphoinositide that is known to regulate many aspects of lysosome function (McCartney et al., 2014). Recent high-resolution structural studies revealed that several amino acid (AA) residues in the selectivity filter of TPCs confer the selectivity of Na^+^ over K^+^ or Ca^2+^ (Guo et al., 2016; Guo et al., 2017), and that PI(3,5)P_2_ binds directly to several positively charged AA residues in the S4-S5 linker to induce channel opening (Kirsch et al., 2018; She et al., 2018; She et al., 2019). Sphingosines also reportedly induce TPC1-mediated Ca^2+^ release from the lysosomes (Höglinger et al., 2015), but direct activation of TPCs by sphingosines was not confirmed in the lysosomal electrophysiological assays (unpublished data in the Xu laboratory) (Li et al., 2019).

Lysosomal membrane potential (Δψ) has been proposed to regulate an array of lysosomal functions, including metabolite transport and membrane trafficking, but the underlying mechanisms are poorly understood (Li et al., 2019; Xu and Ren, 2015). Na^+^ flux mediated by TPCs may cause rapid changes of lysosomal Δψ , which may in turn modulate the functions of TPCs and other lysosomal channels (Cang et al., 2014). Like canonical voltage-gated cation channels, TPCs contain multiple positively-charged AA residues in their voltage sensor domains (S4), which are believed to confer voltage-dependent activation of plant and animal TPC1 channels (Cang et al., 2014; She et al., 2019). In a sharp contrast, TPC2 activation is completely voltage-independent, despite the presence of multiple Arginine/Lysine residues in their S4 helices (Cang et al., 2014; Cang et al., 2013; Wang et al., 2012). In the current study, we identified seven small molecules known to act as tri-cyclic anti-depressants (TCAs) that activate both TPC1 and TPC2 in a voltage-dependent manner.

## Results

### High-throughput screening of small-molecule agonists of TPC2 channels

**Figure 1.**
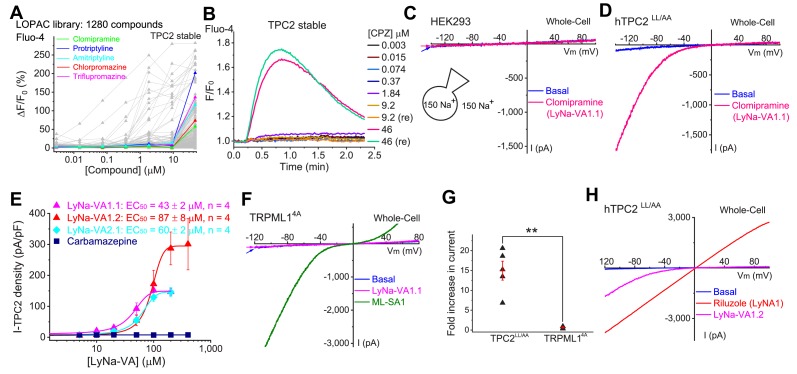
Screening of small-molecule agonists of TPC2. (**A**) High-throughput screening of the LOPAC library with Fluo-4 Ca^2+^ imaging in HEK293 cells stably expressing hTPC2 (Dryad, http://doi.org/10.5061/dryad.s5f6j9h). Each trace represented the average Ca^2+^ response to individual LOPAC compound. Only positive hits confirmed with electrophysiology were color-coded. (**B**) An example of a positive responder (chlorpromazine, CPZ), which elevated intracellular Ca^2+^ levels at the concentration of 46 µM. Note a similar response was seen with a repeated (re) drug application. (**C**) Representative whole-cell currents in a HEK293 cell upon bath application of clomipramine (100 µM). Both pipette and bath solutions contained symmetric 150 mM Na^+^. Currents were elicited by repeated voltage ramps (−140 to +100 mV; 200 ms) with a 4 s inter-step interval. Holding potential (HP) = 0 mV. (**D**) Representative TPC2-mediated currents (*I*_TPC2_) activated by clomipramine (100 µM; LyNa-VA1.1) in a HEK293 cell transfected with a surface-expressing mutant TPC2 channel (EGFP-TPC2^LL/AA^; Wang et al., 2012). (**E**) Dose-dependent activation of TPC2 by Lysosomal Na^+^ channel Voltage-dependent Activators (LyNa-VAs). (**F**) The effect of clomipramine (100 µM) on surface-expressing mutant TRPML1 channels (TRPML1^4A^; Shen et al., 2012). (**G**) Summary of clomipramine effects on whole-cell *I*_TPC2-LL/AA_ and *I*_TRPML1-4A_. Individual data and Mean ± S.E.M are presented. **, p<0.01. (**H**) Activation of *I*_TPC2_ by LyNa-VA1.2 and Lysosomal Na^+^ channel Agonist 1 (LyNA1; see Figure 1—figure supplement 2). Individual data for (**A**), (**E**) and (**G**) are presented in Figure 1—source data 1. Figure 1—source data 1.Screening of small-molecule agonists of TPC2.

We recently used a Ca^2+^-imaging-based high-throughput screening (HTS) method to identify small-molecule agonists for lysosomal TRPML1 channels (Wang et al., 2015). Although TPCs are Na^+^-selective channels with limited Ca^2+^ permeability (Cang et al., 2014; Guo et al., 2017; Li et al., 2019; Wang et al., 2012), in a number of cell-based studies, TPCs reportedly mediate Ca^2+^ release from lysosomes (Brailoiu et al., 2009; Calcraft et al., 2009; Jha et al., 2014; Morgan et al., 2015; Patel, 2015; Ruas et al., 2015; Zong et al., 2009), and it is conceivable that the small Ca^2+^-permeability of TPCs, or activation of Na^+^-dependent Ca^2+^ flux mechanisms (e.g., Na^+^-Ca^2+^ exchanger) secondary to Na^+^ flux may be sufficient to elevate intracellular Ca^2+^. We thus screened HEK293 cells stably expressing human TPC2 (hTPC2) channels with the Library of Pharmacologically Active Compounds (LOPAC) (Liu et al., 2010), the same library of chemicals that were previously tested on TRPML1 channels. Among the positive hits, 23 compounds induced Ca^2+^ increases in TPC2 stable cells (Figure 1A and B and Figure 1—figure supplement 1A), but not in cells stably expressing TRPML1^4A^ (a surface-expressing mutant TRPML1 [Shen et al., 2012]) channels (data not shown).

**Figure 1—figure supplement 1.**
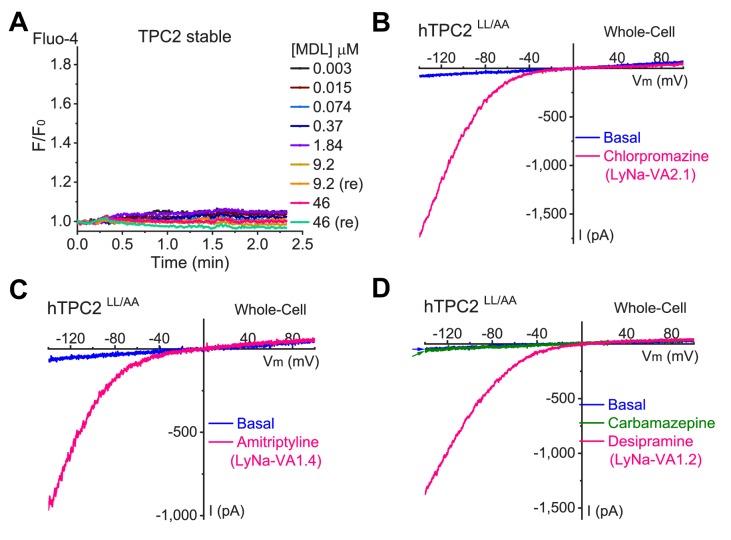
TCAs act as TPC agonists. (**A**) An example of a negative responder (MDL) in high- throughput screening of the LOPAC library with Fluo-4 Ca^2+^ imaging in HEK293 cells stably expressing hTPC2. (**B, C, D**) Representative whole-cell *I*_TPC2_ was evoked by chlorpromazine (100 µM, (**B**), amitriptyline (100 µM, (**C**), and desipramine (100 µM, (**D**) and, but not by carbamazepine (100 µM, (**D**).

**Figure 1—figure supplement 2.**
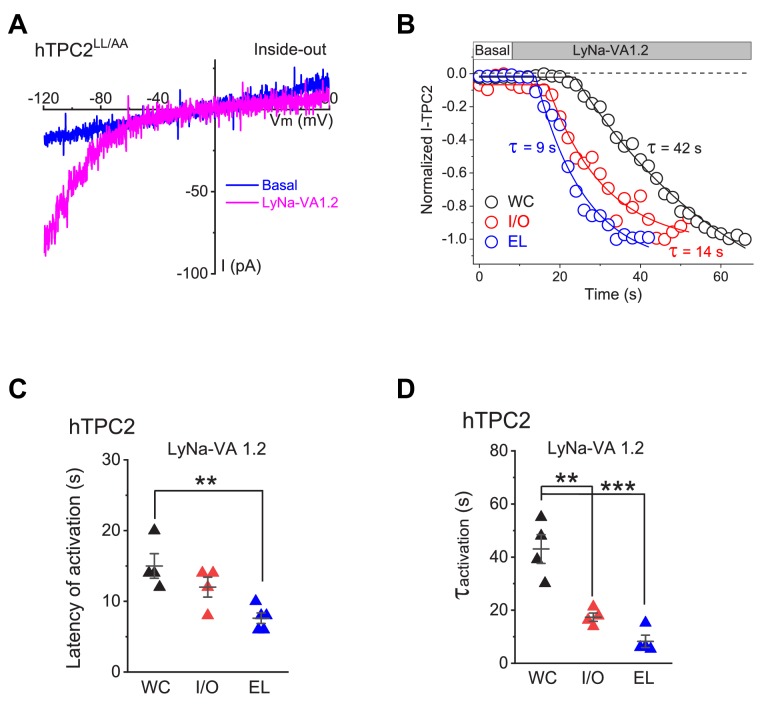
Time courses of LyNa-VA1.2-induced TPC2 activation under different recording configurations. (**A**) Representative *I*_TPC2_ evoked by LyNa-VA1.2 (200 µM) in an inside-out patch from TPC2^LL/AA^-transfected cells. (**B**) Representative time course of LyNa-VA1.2- induced TPC2 activation under whole-cell (WC), inside-out (I/O), and whole-endolysosomal (EL) configurations. (**C, D**) Comparison of latency (**C**) and time constant (τ, **D**) of LyNa-VA1.2 (200 µM)-induced TPC2 activation in whole-cell (latency = 15 ± 2 s, τ = 43 ± 5 s, n = 4), inside-out (latency = 12 ± 1 s, τ = 17 ± 2 s, n = 4), and whole-endolysosome (latency = 7 ± 1 s, n = 5; τ = 8 ± 2 s, n = 4) recordings. Both individual data points and Mean ± S.E.M are presented. **, p<0.01; ***, p<0.001. Individual data for (**B–D**) are presented in Figure 1—figure supplement 2—source data 1. Figure 1—figure supplement 2—source data 1.Time course of LyNa-VA1.2- induced *I*_TPC2_ .

**Figure 1—figure supplement 3.**
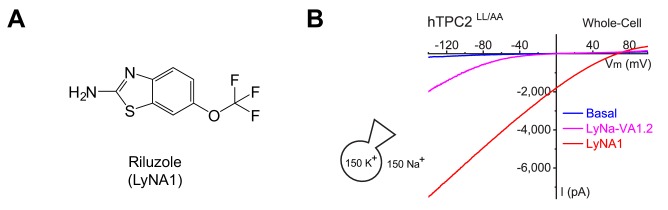
LyNA1 activates TPC2 channels. (**A**) Chemical structure of LyNA1. (**B**) The effects of LyNa-VA1.2 and LyNA1 on whole-cell *I*_TPC2_ under the recording conditions of extracellular 150 mM Na^+^ and cytosolic 150 mM K^+^.

### Tri-cyclic anti-depressants (TCAs) as TPC2 agonists

To our surprise, 5 out of the 23 compounds are well characterized as tri-cyclic anti-depressants (TCAs), which are believed to act on neurotransmitter transporters or voltage-gated Na^+^ channels (Cheng and Bahar, 2019; Pancrazio et al., 1998). We therefore characterized the responses of TPC2 channels to TCAs in detail using electrophysiological methods. We first performed whole-cell recordings in HEK293 cells that were transfected with surface-expressed TPC2 mutant channels (TPC2-L^11^L^12^-AA; abbreviated as TPC2^LL/AA^ hereafter). No functional difference was noted between wild-type (WT) TPC2 and TPC2^LL/AA^ channels in terms of channel permeation and gating properties (Wang et al., 2012). To facilitate the detection of TPC2-specific currents, we used symmetric Na^+^ solutions in the bath (extracellular) and pipette (cytosolic) solutions (Wang et al., 2012). All five TCAs robustly and rapidly activated whole-cell currents in TPC2^LL/AA^-expressing cells (Wang et al., 2012), but not in non-transfected HEK293 cells (Figure 1C and D, Figure 1—figure supplement 1B–D, Table 1, and Table 1—source data 1).

**Table 1.** Summary of electrophysiology-based screening of TPC agonists. Based on chemical structures, LyNa-VAs were divided into two groups: LyNa-VA1.x and LyNa-VA2.x. EC_50_ and the average TPC2 currents (*I*_TPC2_) were calculated based on 3–5 whole-cell or whole-endolysosome recordings (see individual data in Table 1—source data 1) for each LyNa-VA, respectively. Table 1—source data 1.Electrophysiology-based screening of TPC agonists.

LyNa-VAs	Chemical name	Structure	EC_50_ (μM)*	*I*_TPC2_ (pA)**
LyNa-VA1.1	Clomipramine	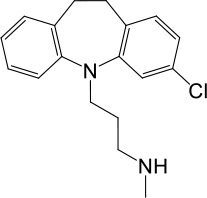	43 ± 2	945 ± 111
LyNa-VA1.2	Desipramine	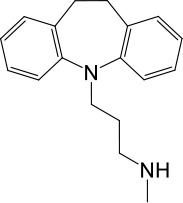	87 ± 8	1120 ± 94
LyNa-VA1.3	Imipramine	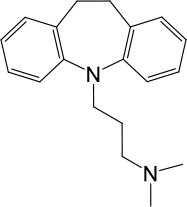	112 ± 1	433 ± 94
LyNa-VA1.4	Amitriptyline	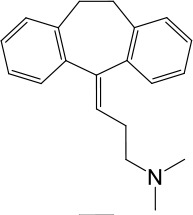	102 ± 3	876 ± 196
LyNa-VA1.5	Nortriptyline	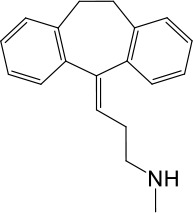	52 ± 10	1916 ± 361
	Carbamazepine	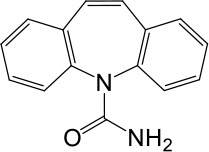	No activation	No activation
LyNa-VA2.1	Chlorpromazine	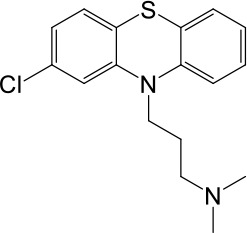	60 ± 2	1101 ± 508
LyNa-VA2.2	Triflupromazine	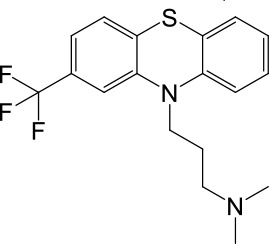	63 ± 2	984 ± 294
	Phenothiazine	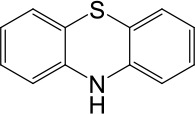	No activation	No activation

^*^Data were obtained from whole-cell recordings at −140 mV. ^**^Data were obtained from whole endolysosome recordings with 100 µM of LyNa-VAs at −120 mV.

We then extended detailed analyses to other known TCAs (Table 1). Of them, Clomipramine activated whole-cell TPC2^LL/AA^-mediated strongly rectifying currents (*I*_TPC2-LL/AA_) with an EC_50_ of 43 ± 2 μM (n = 4 patches) (Figure 1D and E, and Table 1). Given the apparent voltage-dependent gating described below, we referred to Clomipramine as Lysosomal Na^+^ channel Voltage-dependent Activator 1.1 (LyNa-VA1.1). Another structurally different TCA, Chlorpromazine, had an EC_50_ of 60 ± 2 μM (n = 4 patches), and was referred to as LyNa-VA2.1 (Figure 1E, Table 1, and Figure 1—figure supplement 1B). Other TPC-activating TCAs were referred to as LyNa-VA1.x or LyNa-VA2.x, respectively, based on the structural similarity (Table 1). In contrast, no significant activation was seen with Carbamazepine or Phenothiazine, tricyclic drugs without the aliphatic chain (Table 1 and Figure 1—figure supplement 1D). All TCA-induced currents exhibited strong voltage-dependence and inward rectification, which resembled TRPML1-mediated currents (*I*_TRPML1_) (Shen et al., 2012). However, none of the TCAs had any activation effect on *I*_TRPML1_ (Figure 1F and G). Taken together, these results suggested that TCAs may function as small-molecule agonists of TPC2 channels.

In a separate screen, we identified another compound (Riluzole, see Figure 1—figure supplement 3A) that showed striking differences from the responses elicited by TCAs. Riluzole, an FDA-approved amyotrophic lateral sclerosis drug that is known to modulate voltage-gated Na^+^ channels (Liu and Wang, 2018), activated large and linear whole-cell currents in TPC2^LL/AA^-expressing HEK293 cells (Figure 1H and Figure 1—figure supplement 3), but not in non-transfected HEK293 cells. Given its lack of the voltage-dependence, Riluzole was referred to as Lysosomal Na^+^ channel Agonist 1 (LyNA1). Hence, more than one agonist-specific gating (voltage-dependent and voltage-independent) mechanism may co-exist within one channel protein.

### TCAs activate lysosomal TPC2 channels

**Figure 2.**
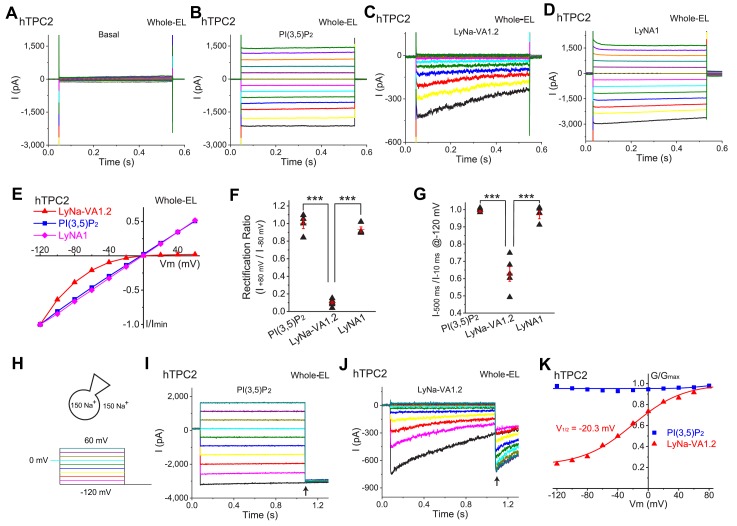
LyNa-VAs activate lysosomal TPC2 channels in a voltage-dependent manner. (**A**) Representative basal *I*_TPC2_ step currents elicited by a voltage step protocol in the whole-endolysosome (EL) configuration. Voltage steps from −140 to 100 mV with a voltage increment (∆V) of 20 mV for 0.5 s were used to elicit *I*_TPC2_ in A-D. HP = 0 mV. Unless otherwise indicated, symmetric (bath/cytosol vs pipette/lumen) Na^+^ (150 mM) solutions were used for all whole-endolysosome recordings, and PI(3,5)P_2_ (0.3 µM), LyNa-VA1.2 (100 µM), and LyNA1 (300 µM) were bath- applied to induce *I*_TPC2_. (**B–D**) Representative *I*_TPC2_ step currents activated by PI(3,5)P_2_ (**B**), LyNa-VA1.2 (**C**), and LyNA1 (**D**). (**E**) Representative normalized I-V plots based on the instantaneous currents activated by various agonists. (**F**) Rectification index, calculated as the ratio of the current amplitudes between +80 and −80 mV, of PI(3,5)P_2_-, LyNa-VA1.2-, and LyNA1- activated *I*_TPC2_. (**G**) The inactivation of *I*_TPC2_ at −120 mV was quantified as the ratio of current amplitudes at 10 *vs.* 500 ms, based on step currents in **B**, (**C**) and D. (**H**) Voltage steps from −120 to 100 mV (∆V = 20 mV) for 1 s were used to elicit tail currents at −120 mV shown in (**I**) and (**J**). (**I**) The tail currents of PI(3,5)P_2_- evoked whole-endolysosome *I*_TPC2_. (**J**) The tail currents of LyNa-VA1.2- activated whole-endolysosome *I*_TPC2_. Arrows in (**I**) and (**J**) indicate where the currents were measured to calculate the channel conductance (G = I/V). (**K**) Normalized G-V curves of PI(3,5)P_2_- and LyNa-VA1.2- activated *I*_TPC2_. LyNa-VA1.2 activated *I*_TPC2_ in a voltage dependent manner with a V_1/2_ = −20.3 ± 3.5 mV (n = 5 patches). For panels **F** and **G**, individual data and Mean ± S.E.M. are presented. ***, p<0.001. Individual data for (**F**) and (**G**) are presented in Figure 2—source data 1. Figure 2—source data 1.The inactivation of *I*_TPC2_ and rectification index of TPC2.

TCAs, with a general structure of an aromatic greasy core and an aliphatic chain containing a terminal amine, are lysosomotropic compounds that are known to be highly accumulating in the lysosomes due to proton trapping (Beckmann et al., 2014). We next tested the effects of LyNa-VAs in hTPC2-transfected Cos1 and HEK293 cells using whole-endolyosome patch-clamp. Cells were pretreated with vacuolin-1 (1 µM) that can selectively increase the size of late endosomes and lysosomes (LELs) up to 5 μm (Dong et al., 2010). The enlarged endolysosomes were manually isolated and then patch clamped in the whole-endolysosome configuration (Dong et al., 2010). In TPC2-positive enlarged LELs isolated from transfected Cos1 cells, little or no basal whole-endolysosome currents were seen under symmetric (pipette/luminal and bath/cytosolic) Na^+^ solutions (Figure 2A). Consistent with our previous studies, bath application of PI(3,5)P_2_, the endogenous agonist of TPCs (Wang et al., 2012), activated a large whole-endolysosome *I*_TPC2_ with linear I-V (Figure 2B and E). Both LyNa-VAs and LyNA1 readily activated *I*_TPC2_, although the I-Vs were dramatically different (Figure 2C, D and E, and Figure 2—figure supplements 1 and 2). In contrast, no current activation was seen for LyNa-VAs or LyNA1 in TRPML1-transfected cells (Figure 2—figure supplement 1C). Both LyNa-VAs and LyNA1 activated whole-endolysosome *I*_TPC_ in WT HAP1 cells, but not HAP1 cells lacking *TPC1* or *TPC2* (*TPC1^−/−^/TPC2^−/−^*, TPC1/2 DKO; Figure 2—figure supplement 1), suggesting that the activation effects of both TCAs and LyNA1 on TPCs are specific. In addition, robust activation of *I*_TPC2_ in the whole-cell configuration with agonists being applied from the extracellular side (analogous to the luminal side in the lysosome), as well as in the inside-out (Figure 1—figure supplement 2A) and whole-endolysosome configurations with agonists being applied from the cytosolic side, suggests that LyNa-VAs and LyNA1 are likely to be membrane permeable, activating TPC2 via direct agonist binding. As the channel activation in the whole-cell recordings was significantly slower, that is longer latency and time course of activation, compared to the inside-out and whole-endolysosome recordings (see Figure 1—figure supplement 2), the action site of LyNa-VAs is likely to be either intracellular or more accessible from the intracellular side.

**Figure 2—figure supplement 1.**
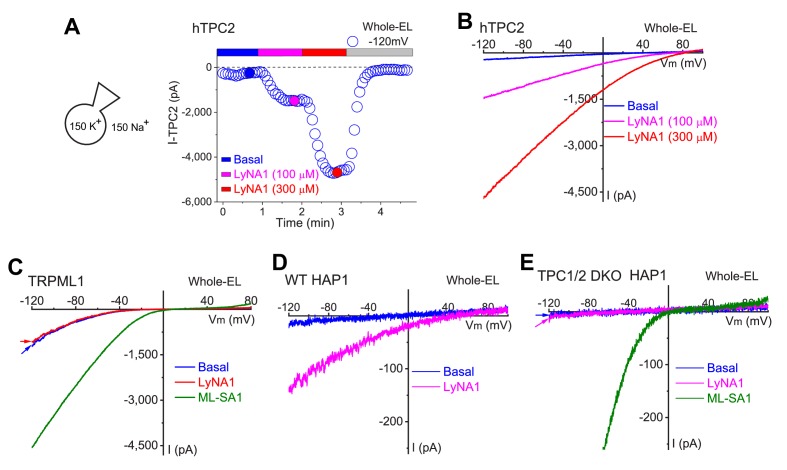
LyNA1 activates lysosomal TPC2 channels. (**A**) Time course of dose-dependent activation of whole-endolysosome *I*TPC2 by LyNA1. (**B**) Representative traces of *I*_TPC2_ evoked by 100 µM and 300 µM LyNA1 at the time points indicated in (**A**). (**C**) The lack of the activation effects of LyNA1 on whole-endolysosome *I*_TRPML1_. (**D, E**) LyNA1 (300 µM) activated whole-endolysosome *I*_TPC_ in WT (**D**) but not TPC1/2 double KO HAP1 cells (**E**). Instead, ML-SA1, a TRPML-specific synthetic agonist, readily activated *I*TRPML1 in TPC1/2 DKO cells.

**Figure 2—figure supplement 2.**
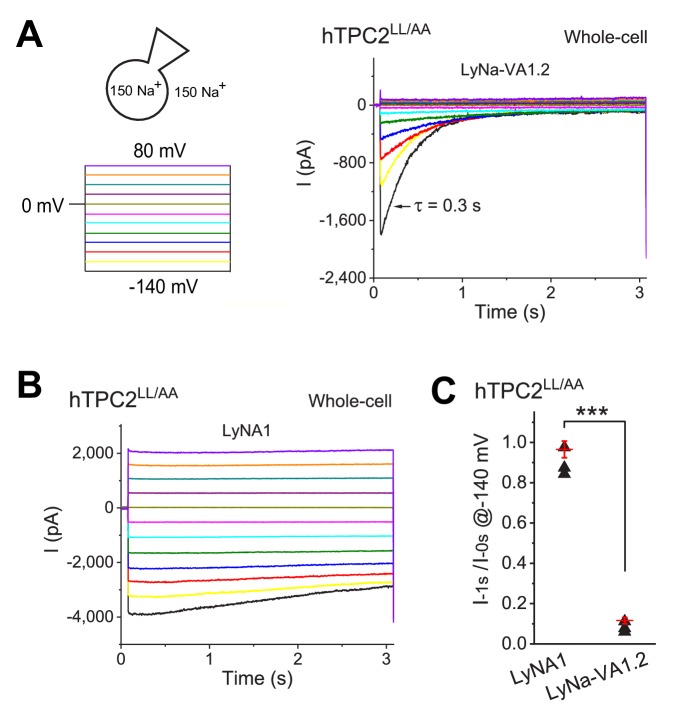
TCAs induce voltage-dependent activation and inactivation of TPC2 channels. (**A, B**) Whole-cell *I*TPC2 elicited by voltage steps from −140 to 80 mV (∆V = 20 mV; see the voltage protocol in the left panel) in the presence of LyNa-VA1.2 (**A**) or LyNA1 (**B**). (**C**) Agonist-specific voltage-dependent inactivation of *I*TPC2 induced by LyNa-VA1.2 and LyNA1 (see individual data in Figure 2—figure supplement 2—source data 1). ***, p<0.001. Figure 2—figure supplement 2—source data 1.Agonist-specific voltage-dependent inactivation of *I*_TPC2_ induced by LyNa-VA1.2 and LyNA1.

### Activation of TPCs by LyNa-VAs shows strong voltage dependence

Despite the fact that TPCs have two S4 domains that act as voltage sensors in many voltage gated channels (She et al., 2018), the responses of TPC2 to PI(3,5)P_2_ and LyNA1 were voltage independent, with large currents at both positive and negative voltages (Figure 2B, D and E). In contrast, TPC2 currents activated by TCAs were strongly voltage-dependent, in both whole-cell and whole-endolysosome recordings, no matter whether the currents were elicited by a voltage ramp or a series of voltage steps (see Figure 2C and E and Figure 2—figure supplement 2). In both whole-cell and whole-endolysosome configurations, TCA-activated step currents displayed prominent inactivation and strong inward rectification (Figure 2C, E and F and Figure 2—figure supplement 2), suggesting the existence of voltage-dependent activation and/or inactivation gating processes. The steady-state voltage dependence of the responses to all 7 TCAs were qualitatively similar. A convenient way to quantitatively summarize the voltage dependence of the TCA responses was to calculate the rectification ratio ($\frac{I@+80mV}{I@-80mV}$ ), which was 1.00 ± 0.06 (n = 4 patches) for activation by PI(3,5P)_2_, 0.93 ± 0.03 for LyNA1 (n = 4), but only 0.10 ± 0.03 for LyNa-VA1.2 (n = 6, Figure 2F).

Under the voltage-step protocols, LyNa-VA-activated *I*_TPC2_ exhibited substantial time-dependent inactivation at negative voltages (Figure 2C and G and Figure 2—figure supplement 2A). This could be conveniently characterized by two parameters: the amount of current decline at steady state ($\frac{I@500ms}{I@10ms}$) and the time constant (τ) of the decay of current. There was no inactivation with ratio near 1.0 at all potentials when the agonist was PI(3,5)P_2_ or LyNA1, but the ratio was 0.6 or less for all the active TCAs tested at −120 mV; the time constant of inactivation was approximately 300 ms at −120 mV (Figure 2J a*nd* Figure 2—figure supplement 2).

When we used a tail-current protocol to study the voltage-dependent activation, the activation by LyNa-VA1.2 was strongly dependent on voltage, with a half-maximal activation voltage (V_1/2_) of −20 mV at the concentration of 100 μM (Figure 2J and K). In contrast, no apparent voltage-dependent activation was seen when whole-endolysosome *I*_TPC2_ was activated by PI(3,5)P_2_ (Figure 2I and K) or LyNA1. Collectively, LyNa-VAs have manifested multiple aspects of voltage-dependent gating of TPC2 channels.

### Synergistic activation of TPC2 by PI(3,5)P_2_ and TCAs

**Figure 3.**
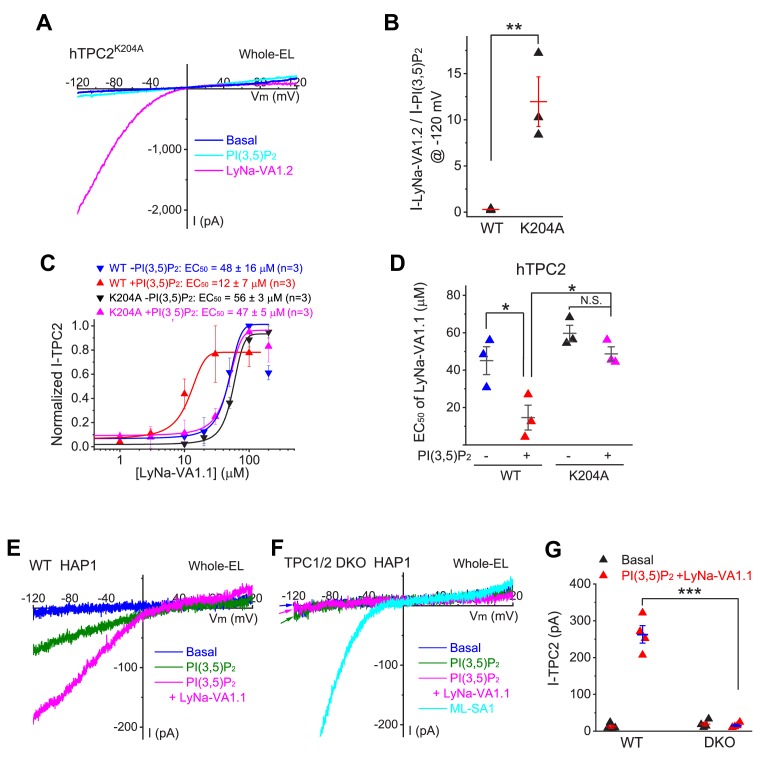
Synergistic activation of TPC2 channels by TCAs and PI(3,5)P_2_. (**A**) The effects of PI(3,5)P_2_ (0.3 µM) and LyNa-VA1.2 (100 µM) on whole-endolysosome *I*_TPC2-K204A_ in TPC2^K204A^-transfected HEK293 cells (She et al., 2019). (**B**) Comparison effects of LyNa-VA1.2 and PI(3,5)P_2_ on WT and PI(3,5)P_2_-insensitive K204A (She et al., 2019) mutant TPC2 channels (also see Figure 3—figure supplement 1). (**C**) The synergistic effects of PI(3,5)P_2_ (50 nM) and LyNa-VA1.1 on whole-endolysosome *I*_TPC2_ and *I*_TPC2-K204A_ currents. Data are presented as Mean ± S.E.M (n = 3 patches). (**D**) The summary of EC_50_ of LyNa-VA1.1 with or without PI(3,5)P_2_ for WT and K204A mutant TPC2 channels. (**E, F**) Co-application of PI(3,5)P_2_ (0.3 µM) and LyNa-VA1.1 (50 µM) activated whole-endolysome *I*TPC in WT (**E**) but not TPC1/2 DKO (**F**) HAP1 cells. Note that the endogenous TPCs were more difficult to activate compared to overexpressed TPCs. (**G**) Summary of LyNa-VA1.1 effects on whole-endolysome *I*_TPC_ in WT and TPC1/2 DKO cells. For panels B, D and F, individual data and Mean ± S.E.M are presented. *, p<0.05; **, p<0.01; ***, p<0.001; N.S., no significance. Individual data for (**B–D**) and (**G**) are presented in Figure 3—source data 1. Figure 3—source data 1.Synergistic activation of TPC2 channels by TCAs and PI(3,5)P_2_.

PI(3,5)P_2_ reportedly binds to Lys204 and adjacent AA residues to activate TPC2 (She et al., 2019). In the cells that were transfected with a PI(3,5)P_2_-insensitive mutant TPC2 channel (K204A) (She et al., 2019), LyNa-VA and LyNA1 still robustly activated whole-endolysosome *I*_TPC2_ (Figure 3A and B a*nd* Figure 3—figure supplement 1A and B). Lysosomal PI(3,5)P_2_ may play a permissive role in TPC activation in intact cells (Ruas et al., 2015). Much dramatic LyNa-VA activation was seen in the presence of a low concentration of PI(3,5)P_2_ (50 nM; Figure 3C and D and Figure 3—figure supplement 1C), and this synergism was nearly abolished in TPC2^K204A^ mutant channels (Figure 3C and D). In contrast, an additive but not synergistic activation was observed between LyNA1 and PI(3,5)P_2_ (Figure 3—figure supplement 1D and E).

**Figure 3—figure supplement 1.**
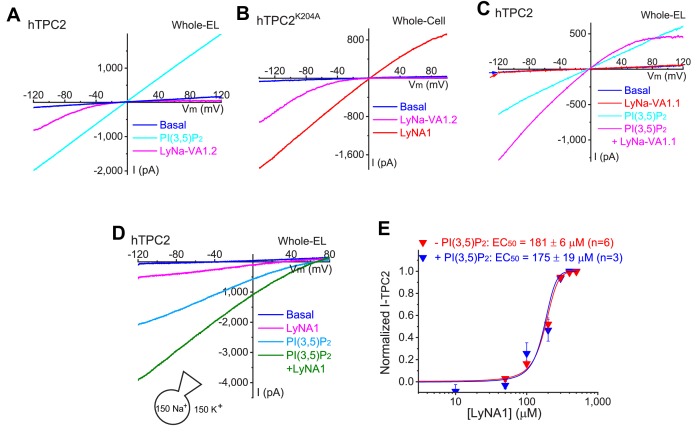
LyNa-VAs and LyNA1 activate TPCs independent of PI(3,5)P_2_. (**A**) The effects of PI(3,5)P_2_ (0.3 µM) and LyNa-VA1.2 (100 µM) on whole-endolysosome *I*TPC2 in TPC2-transfected Cos1 cells. (**B**) Whole-cell *I*TPC2-K204A activated by LyNa-VA1.2 and LyNA1 in TPC2^K204A^-transfected HEK293 cells. (**C**) Synergetic effects of PI(3,5)P_2_ (50 nM) and LyNa-VA1.1 (30 µM) on whole-endolysosome *I*TPC2. (**D**) Additive effects of PI(3,5)P_2_ (50 nM) and LyNA1 (150 µM) on whole-endolysosome *I*TPC2. (**E**) Dose-dependent activation of TPC2 by LyNA1 in the presence or absence of PI(3,5)P_2_ (10 nM). Both individual data points and Mean ± S.E.M are presented (n ≥ 3 patches; also see individual data in Figure 3—figure supplement 1—source data 1). Figure 3—figure supplement 1—source data 1.Dose-dependent activation of TPC2 by LyNA1 in the presence or absence of PI(3,5)P_2_.

Although LyNa-VAs weakly activated endogenous TPC currents, in the presence of PI(3,5)P_2_, more robust activation was seen (Figure 3E and G). In contrast, no measurable whole-endososome *I*_TPC_ was seen in TPC1/2 DKO HAP1 cells even in the presence of both PI(3,5)P_2_ and LyNa-VAs (Figure 3F and G). Collectively, these results suggested that LyNa-VAs activated or modulated TPC2 via a unique, PI(3,5)P_2_-independent but voltage-dependent mechanism.

### Voltage-dependent activation of TPC1 by TCAs

**Figure 4.**
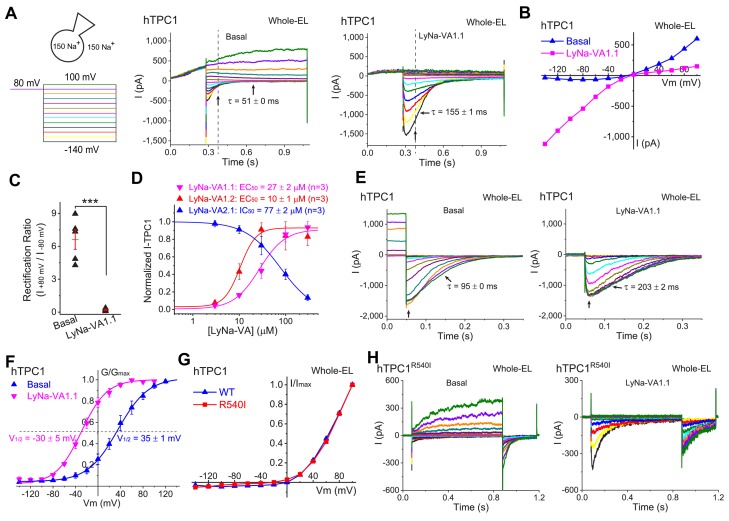
Activation of lysosomal TPC1 channels by LyNa-VAs. (**A**) Whole-endolysosome TPC1 current (*I*_TPC1_) was activated by LyNa-VA1.1 (right) and elicited by a voltage step protocol (left), in which a preconditioning voltage (80 mV, 0.3 s) was applied before voltage steps starting from −140 to 100 mV (0.8 s, ∆V = 20 mV). HP = 0 mV. Unless otherwise indicated, symmetric 150 mM Na^+^ solutions were used for all whole-endolysosome recordings, and LyNa-VA1.1 (100 µM) was bath-applied. (**B**) I-V plots of basal- and LyNa-VA1.1-induced *I*_TPC1_, which were recorded from the same vacuole. Dotted lines in A indicate where the currents were measured. (**C**) Summary of rectification index of basal and LyNa-VA1.1-induced *I*_TPC1_. Individual data and Mean ± S.E.M are presented. ***, p<0.001. (**D**) Does-dependent activation or inhibition of TPC1 by LyNa-VA1.1, LyNa-VA1.2, and LyNa-VA2.1. Data are presented as Mean ± S.E.M (n = 3 patches). (**E**) The effects of LyNa-VA1.1 on *I*_TPC1_ tail currents at −120 mV. The voltage protocol that elicited *I*_TPC1_ tail currents was shown in Figure 4—figure supplement 1A. (**F**) The effects of LyNa-VA1.1 on the G-V curves of *I*_TPC1_ (n = 4–5 patches). (**G**) Normalized I-V plots of basal voltage-dependent currents in WT TPC1- and TPC1^R540I^-transfected cells. (**H**) The basal and LyNa-VA1.1-activated *I*_TPC1-R540I_ step currents, which were recorded from the same vacuole. Individual data for (**C**), (**D**) and (**F**) are also presented in Figure 4—source data 1. Figure 4—source data 1.Activation of lysosomal TPC1 channels by LyNa-VAs.

In contrast to TPC2, which produces little or no current at any potential under basal conditions, yet a large, voltage-independent conductance increases in the presence of PI(3,5)P_2_, TPC1 shows substantial voltage-dependent currents in the absence of any exogenous agonist (Cang et al., 2014; Guo et al., 2016). It was therefore of interest to explore whether TCAs/LyNa-VAs could also act on TPC1. To investigate this, we used two different voltage paradigms. First, when membrane voltage was stepped to various test potentials after a prolonged pre-pulse to +80 mV and the currents were compared to the basal currents, LyNa-VA1.1 produced a profound suppression of outward currents at potentials positive to E_rev_ (~0 mV), and a substantial enhancement of inward currents negative to E_rev_ (Figure 4A and B). Hence, upon LyNa-VA1 activation, the I-V of TPC1 completely reversed from strong outward rectification to strong inward rectification (Figure 4B and C). Intriguingly, unlike LyNa-VA1.1 and LyNa-VA1.2, which activated inward *I*_TPC2_ with EC_50_s within micromolar ranges, both LyNa-VA2.1 and LyNA1 inhibited *I*_TPC1_ (Figure 4D and Figure 4—figure supplement 1C). Second, we used a tail current paradigm to measure the amplitude of the peak inward current at −120 mV following prolonged steps to various potentials (Figure 4E). The effect of LyNa-VA1.1 was to shift the midpoint of activation voltage (V_1/2_) by −65 mV as compared to the basal condition, and to slow down the deactivation time course (Figure 4E–F).

**Figure 4—figure supplement 1.**
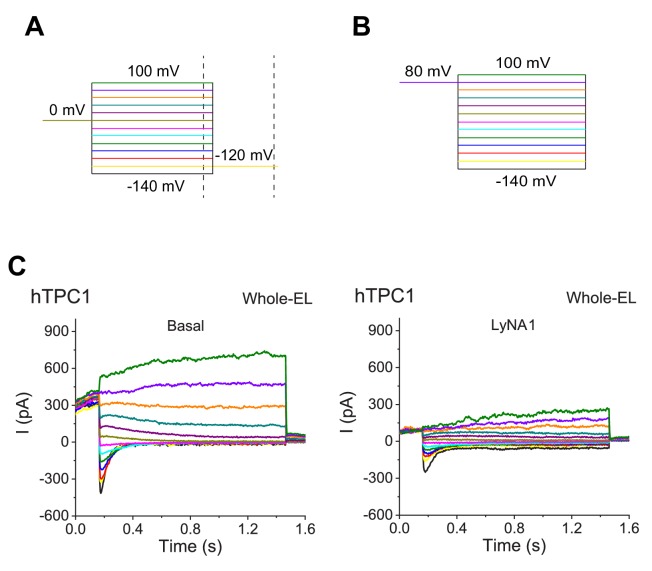
Inhibition of lysosomal TPC1 channels by LyNA-1. (**A**) A voltage protocol that elicited *I*_TPC1_ tail currents as shown in Figure 4E (from −140 to 100 mV with a ∆V = 20 mV, 1 s with a 10 s inter-step interval). Arrows indicate where tail currents were measured. (**B**) A voltage protocol that elicited *I*_TPC1_ tail currents as shown in C. A preconditioning voltage (+80 mV, 0.2 s) was applied before voltage steps starting from −140 to 100 mV (1.3 s, ∆V = 20 mV). HP = 0 mV. (**C**) In comparison to the basal *I*_TPC1_ (right), LyNA1 (150 µM) inhibited (left) *I*_TPC1_.

**Figure 4—figure supplement 2.**
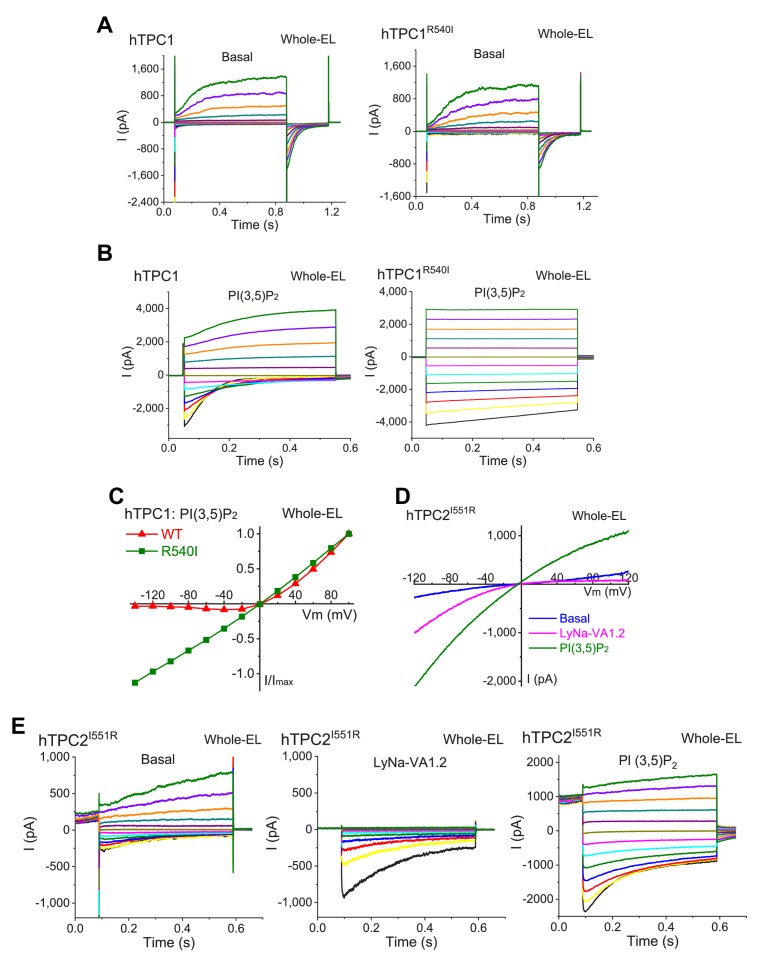
Voltage-dependence and PI(3,5)P_2_-sensitivity of R540I mutant TPC1 channels. (**A**) Basal whole-endolysosome *I*_TPC1_ (left) or *I*_TPC1-R540I_ (right) was elicited by voltage steps (−140 to +100 mV with ΔV = 20 mV) in the absence of PI(3,5)P_2_ in TPC1-transfected Cos1 cells (She et al., 2019). Tail currents were elicited at −120 mV. (**B**) The effects of PI(3,5)P_2_ (0.3 µM) on *I*_TPC1_ (left) and *I*_TPC1-R540I_ (right) step currents. (**C**) I-V plots of steady-state *I*_TPC1_ and *I*_TPC1-R540I_ step currents shown in B. (**D**) LyNa-VA1.2-induced whole-endolysosome *I*_TPC2-I551R_ was evoked by a voltage ramp from −120 to +120 mV (duration = 200 ms). (**E**) The basal (right), LyNa-VA1.2 (200 µM)- (middle) and PI(3,5)P_2_ (0.3 µM)- (left) activated lysosomal *I*_TPC1-I551R_ were recorded from the same vacuole. A preconditioning voltage (+80 mV, 0.1 s) was applied before voltage steps starting from −140 to 100 mV (0.5 s, ∆V = 20 mV). HP = 0 mV.

The S4 segments of TPC1 and TPC2 contain several positively-charged AA residues, which were believed to serve as voltage sensors in mediating TPC1- but not TPC2-specific activation (She et al., 2018). It was recently reported that the R540I mutation, which removes a positive charge in the second putative voltage-sensing ‘S4-type’ helix, abolished TPC1 activation by membrane depolarization (She et al., 2018). However, in our hands, depolarization still robustly activated voltage-dependent currents in the whole-endolysosomes of hTPC1^R540I^ -expressing cells (Figure 4G and H). When LyNa-VA1.1 was tested on endolysosomes overexpressing TPC1^R540I^, there was also a dramatic enhancement of inward currents at negative potentials indicative of a substantial negative shift in the V_1/2_ of voltage activation (Figure 4H). Finally, when exposed to PI(3,5)P_2_, *I*_TPC1_ showed a large enhancement at negative potentials indicative of a negative shift in the V_1/2_ of activation, while TPC1^R540I^ -positive endolysosomes showed large currents at all potentials (Figure 4—figure supplement 2A, B and C). The charge-introducing mutation at Ile551 of TPC2 (She et al., 2019), the equivalent site of TPC1^R540^, conferred a voltage-dependence to the mutant channel but failed to affect LyNa-VA1.2 activation (Figure 4—figure supplement 2D and E). Put together, these results suggested that whereas the S4 voltage-sensing domains may play a modulatory role, there exist intrinsic or extrinsic voltage-sensing mechanisms elsewhere responsible for the voltage-dependent gating of TPCs.

### Cationic ion selectivity of TPC2 is not altered by LyNa-VAs and LyNA1

**Figure 5.**
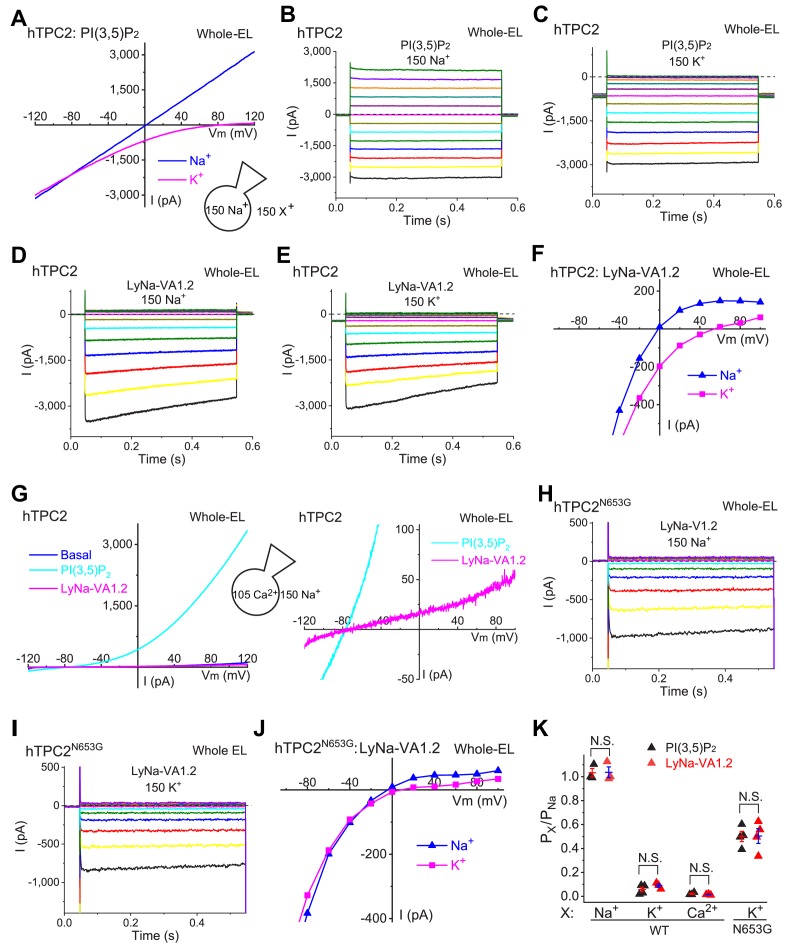
LyNa-VAs do not change the cationic selectivity of TPC2 channels. (**A**) Representative PI(3,5)P_2_-evoked whole-endolysosome *I*_TPC2_ elicited by a voltage ramp from −120 to 120 mV. The recordings were performed under a bi-ionic condition with 150 (in mM) Na^+^ in the pipette solution and 150 Na^+^ or 150 K^+^ in the bath solution. PI(3,5)P_2_ (0.3 µM) was bath-applied. (**B, C**) Representative PI(3,5)P_2_- evoked *I*_TPC2_ elicited with voltage steps (−140 mV to 100 mV with a ∆V = 20 mV, 0.5 s) with 150 mM Na^+^ (**B**) or K^+^(**C**) in the bath solution. (**D, E**) Representative LyNa-VA1.2-activated *I*_TPC2_ step currents. (**F**) Representative I-V plots of LyNa-VA1.2- activated *I*_TPC2_ measured from the instantaneous currents in (**D**) and (**E**). Note the reversal potentials (E_rev_) of LyNa-VA1.2- activated *I*_TPC2_ in the presence of Na^+^ or K^+^ bath solution. (**G**) LyNa-VA1.2- activated *I*_TPC2_ under the bi-ionic conditions of bath/cytosolic Na^+^ and pipette/luminal Ca^2+^. 150 Na^+^ solution contained (in mM) 145 NaCl, 5 NaOH, 20 HEPES, (pH 7.2); isotonic (105 mM) Ca^2+^ solution contained (in mM) 100 CaCl_2_, 5 Ca(OH)_2_, 20 HEPES (pH 7.2). Right panel zoom-in micrograph shows the E_rev_ of LyNa-VA1.2- activated *I*_TPC2_. (**H–J**) Representative I-V plots of LyNa-VA1.2- evoked *I*_TPC2-N653G_ (**J**) measured from Na^+^ (**H**) and K^+^ (**I**) bath solution. (**K**) Summary of Na^+^ *vs.* K^+^ /Ca^2+^ selectivity of WT TPC2 and TPC2^N653G^ channels. Individual data and Mean ± S.E.M are presented (also see Figure 5—source data 1). N.S., no significance. Figure 5—source data 1.Ionic selectivity of WT TPC2 and N653G mutant channels.

The selectivity filter region in an ion channel is responsible for the selective permeability to one or more ions (Yu and Catterall, 2004). However, it was recently reported that AA residues below the selectivity filter may mediate activation gating of multiple K^+^ channels by small-molecule agonists (Schewe et al., 2019). In addition, some voltage sensitivity can be conferred when the permeable ions move through the selectivity filter (Schewe et al., 2016). We therefore tested whether TCAs/LyNa-VAs bind to the selectivity filer. Like PI(3,5)P_2_-activated *I*_TPC2_ (Figure 5A–C and K), LyNa-VA1.2- and LyNA1-induced whole-endolysosome *I*_TPC2_ was highly selective for Na^+^ over K^+^, as substitution of bath/cytoplasmic Na^+^ with K^+^ significantly shifted E_rev_ to more positive values (Figures 1H, 5D–F and K and Figure 2—figure supplement 1B). Meanwhile, LyNa-VA1.2- and LyNA1-activated *I*_TPC2_ still exhibited low Ca^2+^ permeability (Figure 5G and K and Figure 5—figure supplement 1A), and the P_Ca_/P_Na_ values were similar to those for PI(3,5)P_2_-activated *I*_TPC2_ (Wang et al., 2012) (Figure 5K **a**nd Figure 5—figure supplement 1B). Finally, the N653G mutation, which is known to significantly increase the relative K^+^ permeability of the TPC2 channel (Guo et al., 2017), did not alter the ability of LyNa-VA1.2 to enhance channel activation, but did result in currents elicited by LyNa-VA1.2 that had a much increased relative permeability to K^+^ (Figure 5I–K). Taken together, these results indicate that the selectivity filter region does not mediate the action of TCAs.

**Figure 5—figure supplement 1.**
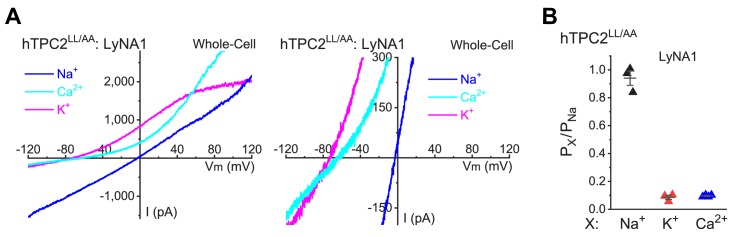
LyNA1 does not change the Na^+^ selectivity of TPC2 channels. (**A**) LyNA1 (300 µM)- activated whole-cell *I*_TPC2-LL/AA_ under the bi-ionic conditions (in mM), that is pipette/cytosolic 150 Na^+^ and bath/extracellular 150 Na^+^, 150 K^+^ or 105 Ca^2+^. Right panel: zoom-in micrograph shows the E_rev_ changes of *I*_TPC2_ with different bath solutions. (**B**) Summary of cationic selectivity values for LyNA1-induced *I*_TPC2_. Both individual data points and Mean ± S.E.M are presented (see individual data in Figure 5—figure supplement 1—source data 1). Figure 5—figure supplement 1—source data 1.LyNA1 does not change ionic selectivity of TPC2 channels.

## Discussion

In this study, we report that the voltage dependence, generally thought to be an intrinsic property of an ion channel (Catterall, 2010; Yu and Catterall, 2004), can be conferred or unmasked by extrinsic agonists in lysosomal TPC channels. What is the origin of agonist-conferred voltage-dependence in the otherwise voltage-independent TPC2 channel? It is possible that the S4 voltage sensors, which are operational in TPC1, might be ‘exposed’ upon agonist binding. Alternatively, another unidentified ‘hidden’ intrinsic voltage sensor might be revealed upon agonist binding, a possibility that was supported by the negative results in our targeted mutational analyses of the S4 regions. Additionally, it is recently reported that permeant ions may contribute to the channel’s voltage-dependence (Schewe et al., 2019). Hence, it is an attractive hypothesis that a gate at the selectivity filter is the molecular target of TCAs to mediate the observed voltage-dependence. However, it remains unknown whether such a ‘selectivity filter gate’, extensively studied in several other ion channels including CNG channels (Contreras et al., 2008), does exist in TPC channels. Nevertheless, although mutational analyses in the selectivity filter or the ‘S6 gate’ (data not shown) do not seem to affect LyNa-VA activation, given the limitations of targeted mutations, future high-resolution structural studies may be necessary to reveal the TCA-binding sites in the TPC channels, explaining the conferred voltage dependence by TCAs. In addition, the differential effects of LyNa-VAs on TPC1 and TPC2 may also help design future structural-functional studies to reveal the action site of TCAs on TPCs.

Diverse functions have been associated with TPC channels, largely based on genetic manipulations (e.g. KO, knockdown, overexpression) (Grimm et al., 2017; Xu and Ren, 2015). However, the roles of TPCs in some of the proposed functions might be indirect based on the following reasons. First, lysosomal membrane trafficking, for example fusion and fission, has been difficult to study as these functions are interconnected. For instance, a block in membrane fusion may often indirectly affect membrane fission, and vice versa (Xu and Ren, 2015). In addition, defects in lysosomal membrane trafficking may also affect lysosomal degradation, and degradation products may in turn regulate membrane trafficking (Xu and Ren, 2015). Second, compensatory changes occur commonly in the genetically-manipulated cells, for example KO or lysosome storage disease (LSD) cells. Hence, it is necessary to develop methods to acutely activate and inhibit lysosomal channels, so that immediate cellular actions of TPC activation can be revealed. Notably, precisely defining TPC’s roles in lysosomal Δψ regulation may require real-time monitoring of lysosomal Δψ while acutely activating or inhibiting lysosomal K^+^ and Na^+^ channels (Li et al., 2019). The identification of membrane-permeable small-molecule TPC agonists has made it feasible for such studies.

TCAs are known to regulate autophagy and lysosome function, but underlying mechanisms are not clear (Tsvetkov et al., 2010). For example, in a neuronal model of Huntington disease (HD), TCAs were shown to be neuroprotective by inducing the clearance of misfolded protein aggregates (Tsvetkov et al., 2010). Given the proposed roles of TPCs in autophagy and lysosomal membrane trafficking (Grimm et al., 2017; Li et al., 2019), it is likely some of the effects associated with TCAs are mediated by TPCs. The concentrations of TCAs that activate autophagy are lower than those activating TPC channels in the current study (Beckmann et al., 2014; Cheng and Bahar, 2019). However, TCAs, as lysosomotropic compounds, are known to accumulate at high concentrations in the lysosomes (Beckmann et al., 2014). In addition, the synergistic effects of TCAs with endogenous ligand, for example PI(3,5)P_2_, suggest that the exposure level of TCAs in the brain might be sufficient to cause robust pharmacological actions, much more potently than the efficacies defined in our channel assays. Future cell biological studies utilizing TCAs with TPC KO as negative controls may confirm whether TCAs modulate autophagy and lysosome function through activation of TPCs.

## Materials and methods

**Key resources table:** 

Reagent type (species) or resource	Designation	Source or reference	Identifiers	Additional information
Cell line (*Homo sapiens*)	HEK293	ATCC	RRID:CVCL_0045	
Cell line (*Homo sapiens*)	HAP1	Horizon Discovery	Cat. #: C631	
Cell line (*Chlorocebus aethiops*)	Cos1	ATCC	RRID:CVCL_0223	
Recombinant DNA reagent	pEGFP-C2-TPC2 (plasmid)	Wang et al., 2012		
Recombinant DNA reagent	pEGFP-C2-TPC1 (plasmid)	Wang et al., 2012		
Sequence-based reagent	TPC1-sgRNA	This paper	sgRNA	CTTGCAGTACTTCAGCACCC
Sequence-based reagent	TPC2-sgRNA	This paper	sgRNA	CCCCAGCGTCGGGCTGCTGC
Sequence-based reagent	TPC1-fw	This paper	PCR primers	ATGGCCCAGACATGTGACTC
Sequence-based reagent	TPC1-re	This paper	PCR primers	TGCCTGTCTCCATCCTCTCA
Sequence-based reagent	TPC2-fw	This paper	PCR primers	TGAGCTGAGCATGAGGCAAG
Sequence-based reagent	TPC2-re	This paper	PCR primers	AAAGGACAAGTGGCCCTGAG
Chemical compound, drug	Desipramin hydrochloride	Sigma	Cat. #: D3900	
Chemical compound, drug	Carbamazepine	Sigma	Cat. #: C4024	
Chemical compound, drug	monensin	Sigma	Cat. #: M5273	
Chemical compound, drug	ionomycin	Sigma	Cat. #: I0634	
Chemical compound, drug	Clomipramine	Cayman Chemical	Cat. #: 15884	
Chemical compound, drug	Imipramine	Cayman Chemical	Cat. #: 15890	
Chemical compound, drug	Amitriptyline	Cayman Chemical	Cat. #: 15881	
Chemical compound, drug	Nortriptyline	Cayman Chemical	Cat. #: 15904	
Chemical compound, drug	Phenothiazine	NCATS	CAS: 92-84-2	
Chemical compound, drug	Triflupromazine	NCATS	CAS: 146-54-3	
Chemical compound, drug	Chlorpromazine	Cayman Chemical	Cat. #: 16129	
Chemical compound, drug	ML-SA1	Princeton BioMolecular Research Inc	Cat. #: OSSK_389119	
Chemical compound, drug	PI(3,5)P_2_	Echelon Biosciences	Cat. #: P-3508	
Chemical compound, drug	vacuolin-1	Calbiochem	Cat. #: 673000	
Software, algorithm	pClamp	pClamp	RRID:SCR_011323	

### Molecular biology

All TPC1 and TPC2 mutants were generated with a site-directed mutagenesis kit (Qiagen) using EGFP- human TPC1 and TPC2 as the templates. All constructs were confirmed by DNA sequencing.

### Mammalian cell lines

Cos1 (ATCC, CRL-1650, passage number 8–15) and HEK293 (ATCC, CRL-1573, passage number 8–15) cells were cultured in DMEM with 10% fetal bovine serum (FBS). HAP1 (Horizon Discovery, human HAP1 parental cell line, passage number 5–15) cells were maintained in IMDM with 10% FBS. Immortalized cell lines (Cos1, HEK293 and HAP1) were cultured following standard tissue culture protocols, and were tested negative for mycoplasma contamination using MycoAlert Mycoplasma Detection Kit Assay (Lonza). HEK293 cells are on the list of frequently misidentified or cross-contaminated cell lines, but were only used for the overexpression experiments. Cells were transfected with Lipofectamine 2000 (Invitrogen). After culture media were refreshed 18–24 hr post-transfection, cells were used for the electrophysiological assays 24–36 hr post-transfection.

### TPC1/TPC2 DKO HAP1 cells

TPC1 and TPC2 CRISPR KO cells were generated in HAP1 cells using the CRISPR/Cas9 system. The TPC1 sequence (5'cttgcagtacttcagcaccc3', TPC1-sgRNA) and TPC2 sequence (5'ccccagcgtcgggctgctgc3', TPC2-sgRNA) were targeted with pSpCas9 (BB)−2A-puro vector (Addgene). HAP1 cells were co-transfected with TPC1 and TPC2-sgRNA expressing vector in the presence of Lipofectamine 2000 (In vitrogen) and selected with 2 μg/ml puromycin for 48 hr. The remaining cells were trypsinized and seeded into 96-well plate after a limiting dilution. When single cell clones were established, their genomic DNAs were extracted and amplified with following primers: TPC1-fw: 5’atggcccagacatgtgactc3’, TPC1-re: 5’ tgcctgtctccatcctctca3’; TPC2-fw: 5’tgagctgagcatgaggcaag3’, TPC2-re: 5’ aaaggacaagtggccctgag3’. The PCR amplicons were then sequenced to confirm the intended genetic disruption. The TPC1/TPC2 DKO cells harboring an insert of one nucleotide (A) in the TPC1 sequence and a deletion of ten nucleotides in the TPC2 sequence, respectively, were used in the present study.

### LOPAC high-throughput screening

Ca^2+^ imaging-based HTS using the Library of Pharmacologically Active Compounds (LOPAC)1280 collection (Sigma) was conducted as described previously (Liu et al., 2010). Briefly, HEK293 cells stably expressing TPC2 and TRPML1 were loaded with a Ca^2+^ detection dye (Fluo-4). The kinetic of Ca^2+^ flux was measured using a kinetic plate reader FDSS-7000 (Liu et al., 2010). The FDSS-7000 had an on-board 1536 pintool that was used to transfer 23 nl of a compound to the assay plate (Liu et al., 2010). All of the compounds were dissolved in 100% DMSO. Hence, transferring 23 nl of a compound to a well containing 5 μl of the culture medium would result in a final concentration of DMSO of ~0.5%. The final concentrations of the LOPAC compounds in each were 0.003, 0.015, 0.074, 0.37, 1.84, 9.2, and 46 μM, respectively. Compounds that were positive in the TPC2 assay, but not in the TRPML1 assay, were considered to be positive hits for potential TPC2 agonists.

### Whole-cell electrophysiology

Whole-cell recordings were performed using pipette electrodes with resistance of 3–5 MΩ. Unless otherwise stated, both pipette and bath solutions contained (in mM): 145 NaOH, 5 NaCl, 20 HEPES, pH 7.2 (adjusted with methanesulfonic acid). All bath solutions were applied via a perfusion system that allowed complete solution exchange within a few seconds. Data were collected using an Axopatch 200A patch clamp amplifier, Digidata 1440, and pClamp 10.2 software (Axon Instruments). Whole-cell currents were digitized at 10 kHz and filtered at 2 kHz. All experiments were conducted at room temperature (21–23°C), and all recordings were analyzed with pClamp 10.2, and Origin 8.0 (OriginLab, Northampton, MA).

### Whole-endolysosome electrophysiology

Endolysosomal electrophysiology was performed in isolated enlarged endolysosomes using a modified patch-clamp method (Dong et al., 2010). Cells were treated with 1 μM vacuolin-1, a lipid-soluble polycyclic triazine that can selectively increase the size of endosomes and lysosomes (Huynh and Andrews, 2005), for at least 1 hr and up to 12 hr. Whole-endolysosome recordings were performed on manually isolated enlarged endolysosomes (Wang et al., 2012). In brief, a patch pipette was pressed against a cell and quickly pulled away to slice the cell membrane. Enlarged endolysosomes were released into a dish and identified by monitoring EGFP-TPC1/2 or the mCherry-TPC1/2 fluorescence. After formation of a gigaseal between the patch pipette and the enlarged endolysosome, capacitance transients were compensated. Voltage steps of several hundred mVs with millisecond duration were then applied to break into the vacuolar membrane. The whole-endolysosome configuration was verified by the re-appearance of capacitance transients after break-in.

Unless otherwise stated, both bath (internal/cytoplasmic) and pipette solutions contained (in mM): 145 NaOH, 5 NaCl, 20 HEPES, pH 7.2 (adjusted with methanesulfonic acid). 150 K^+^ solution contained (in mM): 145 KOH, 5 KCl, 20 HEPES, pH 7.2 (adjusted with methanesulfonic acid). Data were collected using an Axopatch 200A patch clamp amplifier, Digidata 1440, and pClamp 10.2 software (Axon Instruments). Whole-endolysosome currents were digitized at 10 kHz and filtered at 2 kHz. All experiments were conducted at room temperature (21–23°C), and all recordings were analyzed with pClamp 10.2, and Origin 8.0 (OriginLab, Northampton, MA). The permeability to cations (relative to P_Na_) was estimated based on following equations (Lewis, 1979) and E_rev_ measurement under bi-ionic conditions:

(1) $$P_{X}/P_{Na}=\gamma_{Na}/\gamma_{X}\;\left\{[Na^{+}{]}_{Luminal}\;/\;[X^{+}{]}_{Cytoplasmic}\right\}\left\{exp(R_{rev}F/RT)\right\}$$

(2) $$P_{Ca}/P_{Na}=\gamma_{Na}/\gamma_{Ca}\;\left\{[Na^{+}{]}_{Cytoplasmic}\;/\;[4[Ca^{2+}{]}_{Luminal}\right\}\left\{exp(E_{rev}F/RT)\right\}\left\{1+exp(E_{rev}F/RT)\right\}$$

where R, T, F, E_rev_, and γ are, respectively, the gas constant, absolute temperature, Faraday constant, reversal potential, and activity coefficient. The liquid junction potentials were measured and corrected as described (Neher, 1992).

### Data analysis

Data are presented as the mean ± standard error of the mean (S.E.M). Statistical comparisons were made using analysis of variance (ANOVA). A p value < 0.05 was considered statistically significant.

## Data Availability

All data generated or analyzed during this study are included in the manuscript and supporting files. Source data files have been provided for: Fig. 1A, 1E, 1G, 2F-G, 3B-D, 3G, 4C-D, 4F, 5K; Table 1; Fig. 1-figure supplement 2, Fig. 2- figure supplement 2C, Fig. 3 -figure supplement 1E and Fig. 5 -figure supplement 1B. LOPAC screening results in Fig. 1A has been deposited in Dyrad: doi: https://doi.org/10.5061/dryad.s5f6j9h. The following dataset was generated: XuHZhangXChenWLiPCalvoRSouthallNHuXBryant-GenevierMFengXGengQGaoCYangMTangKFerrerMMaruganJ2019Data from: Agonist-specific Voltage-dependent Gating of Lysosomal Two-pore Na + ChannelsDyrad Digital Repository10.5061/dryad.s5f6j9hPMC690585531825310

## Additional files

Transparent reporting form
